# Beyond Vocal Nodules: Bilateral Internal Jugular Phlebectasia Presenting With Progressive Dysphonia in an Adult Woman

**DOI:** 10.7759/cureus.110251

**Published:** 2026-06-04

**Authors:** Miguel A Peraza-Arjona, Victor M Ayuso-Diaz, Alfonso Peraza-Fernandez

**Affiliations:** 1 Angiology, Vascular and Endovascular Surgery, Regional Hospital “Elvia Carrillo Puerto” ISSSTE, Yucatán, MEX; 2 Research and Education, Medical Care and Research, Yucatan, MEX; 3 Genomic-Metabolic Unit, University Marista of Merida, Yucatan, MEX; 4 Research and Education, Clínica Yucatán, Yucatan, MEX; 5 Angiology, Vascular and Endovascular Surgery, Regional Hospital "Elvia Carrillo Puerto" ISSSTE, Yucatán, MEX

**Keywords:** bilateral presentation, case report, cervical mass, computed tomography angiography, doppler ultrasound, internal jugular vein ectasia, jugular phlebectasia, valsalva maneuver, vascular anomaly

## Abstract

Jugular phlebectasia is a rare vascular anomaly characterized by a congenital, benign, fusiform dilation of the jugular vein, typically presenting as a soft, non-pulsatile cervical swelling that becomes evident during maneuvers that increase intrathoracic pressure. Although most cases are asymptomatic and diagnosed during childhood, adult presentations - particularly bilateral involvement - remain exceptionally uncommon and may pose a diagnostic challenge due to their resemblance to other cervical masses. When symptomatic, patients may report dysphonia, dysphagia, local discomfort, or cosmetic concerns, depending on the degree of venous dilitation and anatomical relationships.

We report the case of a 52-year-old woman who presented with a long-standing history of progressive dysphonia and intermittent cervical discomfort, initially attributed to laryngeal nodules. Over time, her symptoms evolved, prompting further evaluation. Physical examination revealed a bilateral, soft, compressible cervical mass that became prominent during the Valsalva maneuver and completely regressed at rest, raising suspicion of a dynamic vascular process. Doppler ultrasonography demonstrated marked fusiform dilation of both internal jugular veins with preserved venous flow and no evidence of thrombosis. These findings were further characterized by CT angiography, which confirmed bilateral jugular phlebectasia without associated vascular malformations, flow disturbances, or extrinsic compression.

Given the absence of complications such as thrombosis, rapid enlargement, or airway compromise, and considering the potential risks associated with surgical intervention, a conservative management approach was adopted. The patient was managed with clinical surveillance and symptom-oriented measures, with stable evolution during follow-up.

This case highlights the importance of a thorough clinical examination and the recognition of dynamic cervical masses, particularly those associated with Valsalva maneuvers, to avoid misdiagnosis and unnecessary invasive procedures. Furthermore, it underscores the critical role of non-invasive imaging modalities, especially Doppler ultrasound and CT angiography, in establishing an accurate diagnosis. The rarity of bilateral jugular phlebectasia in adults makes this report a valuable contribution to the literature, reinforcing awareness of this benign condition and supporting conservative management in appropriately selected patients.

## Introduction

Jugular phlebectasia is a rare vascular anomaly characterized by a benign, fusiform, and non-tortuous dilatation of the internal jugular vein [1]. Although its exact etiology remains unclear, it has been associated with congenital weakness of the venous wall, structural abnormalities of connective tissue, and hemodynamic factors that lead to increased venous distensibility [2]. Clinically, it typically presents as a soft, painless, and compressible cervical mass that becomes more prominent during maneuvers that increase intrathoracic pressure, such as coughing or the Valsalva maneuver, which represents a key diagnostic feature [3].

Most cases are diagnosed during childhood and are more frequently observed in males, with a clear predominance of unilateral right-sided involvement [4]. Bilateral involvement is exceptionally rare, representing only a small fraction of reported cases, which contributes to its underrecognition and potential misdiagnosis [2]. Due to its dynamic clinical behavior, this condition may mimic other cervical pathologies that present with Valsalva-dependent swelling, such as laryngocele or pharyngocele, as well as other cervical masses including branchial cleft cysts and superior mediastinal cysts, making differential diagnosis essential [5].

The diagnosis of jugular phlebectasia relies on a high index of clinical suspicion, particularly when evaluating intermittent cervical masses. Non-invasive imaging modalities play a central role, with Doppler ultrasonography serving as the initial diagnostic tool due to its ability to demonstrate venous dilatation, compressibility, and flow characteristics [6]. CT angiography provides further anatomical delineation and confirms the extent of the lesion, allowing exclusion of alternative vascular or structural abnormalities [1]. The increasing availability of advanced imaging techniques has contributed to a higher detection rate of this condition in recent years [1].

Despite being considered a benign entity, there is no universally established management guideline. Current evidence supports a conservative approach in asymptomatic patients, reserving surgical intervention for cases with significant symptoms, cosmetic concerns, or complications such as thrombosis [3]. Awareness of this condition is also relevant in surgical and interventional settings, as anatomical variations of the internal jugular vein may increase the risk of procedural complications [7].

In this report, we present a case of bilateral jugular phlebectasia in an adult woman, an uncommon presentation of this condition, highlighting its dynamic clinical features, imaging correlation, and the rationale supporting a conservative management approach.

## Case presentation

We present the case of a 52-year-old woman, a preschool teacher, with no relevant history of vascular disease or connective tissue disorders, who initially developed symptoms eleven years prior to presentation, characterized by mild dysphagia and progressive dysphonia throughout the day. At that time, laryngoscopic evaluation revealed vocal cord nodules, which were considered the underlying cause of her symptoms, and conservative management was initiated.

Over the following years, the patient experienced recurrence and gradual progression of symptoms, including alterations in voice tone and volume, vocal fatigue, persistent dysphonia, and frequent throat clearing. This evolving clinical pattern was not entirely consistent with the initial diagnosis of vocal cord nodules, raising suspicion of an alternative structural or functional etiology beyond the larynx. Although a direct causal relationship between the jugular phlebectasia and the patient's dysphonia could not be definitively established, the persistence and progression of vocal symptoms despite conservative management for vocal cord nodules raised suspicion of an alternative contributing factor, prompting further diagnostic evaluation.

During a subsequent otolaryngological evaluation, a focused cervical examination was performed. At rest, the neck contour appeared normal; however, during the Valsalva maneuver, a bilateral, soft, painless, compressible, and expansile cervical mass became evident. The swelling was more prominent on the right side, with a sac-like appearance along the anterior border of the sternocleidomastoid muscle. The patient reported a mild sensation of cervical pressure during the maneuver, without associated pain. The masses completely regressed at rest. No signs of airway compromise, mechanical dysphagia, or sensory deficits were identified (Figure 1).

**Figure 1:**
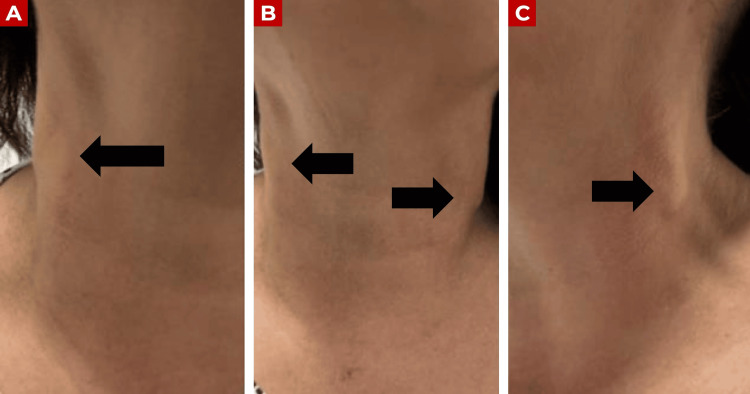
Dynamic clinical appearance of bilateral cervical swelling during Valsalva maneuver Clinical examination of the neck demonstrating a soft, non-pulsatile, and compressible cervical swelling that becomes evident during the Valsalva maneuver. (A) Right-sided cervical region during Valsalva maneuver. The black arrow indicates the focal expansile, swelling with dilatation of the right internal jugular vein along the anterior border of the sternocleidomastoid muscle. (B) Neck at rest. The black arrows indicate the absence of visible cervical swelling in both jugular regions. (C) Left-sided cervical region during Valsalva maneuver. The black arrow indicates a less pronounced expansile swelling with dilatation of the left internal jugular vein.

These findings prompted further imaging evaluation. Doppler ultrasonography of the neck (Figure 2) demonstrated marked fusiform dilation of the right internal jugular vein, measuring 2.4 × 1.6 cm, and a lesser dilation of the left internal jugular vein measuring 1.3 × 1.0 cm. Both vessels exhibited preserved venous flow without evidence of mural thrombosis or abnormal vascular channels. Dynamic assessment during the Valsalva maneuver confirmed exaggerated venous distensibility, consistent with jugular phlebectasia.

**Figure 2:**
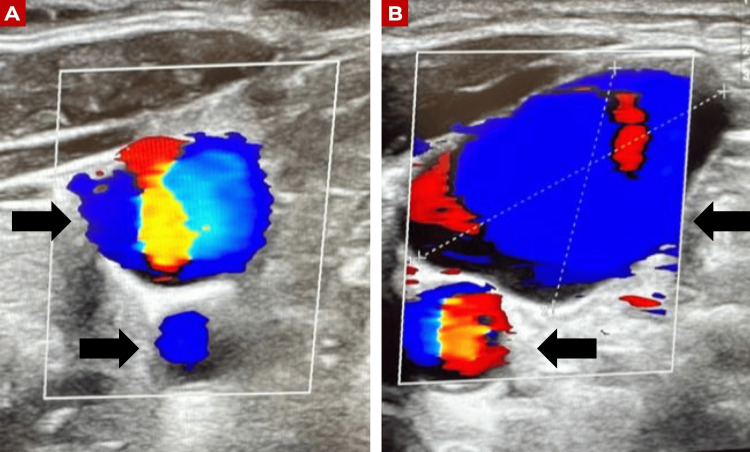
Doppler ultrasonography demonstrating bilateral internal jugular vein phlebectasia Doppler ultrasound of the neck revealing fusiform dilation of both internal jugular veins with preserved venous flow. (A) Transverse Doppler ultrasound of the left cervical region. The upper black arrow indicates the dilated left internal jugular vein, while the lower black arrow indicates the adjacent common carotid artery. (B) Transverse Doppler ultrasound of the right cervical region. The upper black arrow indicates the markedly dilated right internal jugular vein, while the lower black arrow indicates the adjacent right common carotid artery.

Given the suspicion of an uncommon bilateral condition, CT angiography of the supra-aortic trunk area with cranial extension was performed. Imaging revealed a well-defined fusiform dilation of the right internal jugular vein, with a maximum diameter of 2.57 cm at the level of the thyroid gland. No filling defects, stenosis, flow disturbances, or arteriovenous malformations were identified. The left side demonstrated a similar but smaller fusiform dilatation. No extrinsic compression, lymphadenopathy, or associated masses were observed (Figure 3).

**Figure 3:**
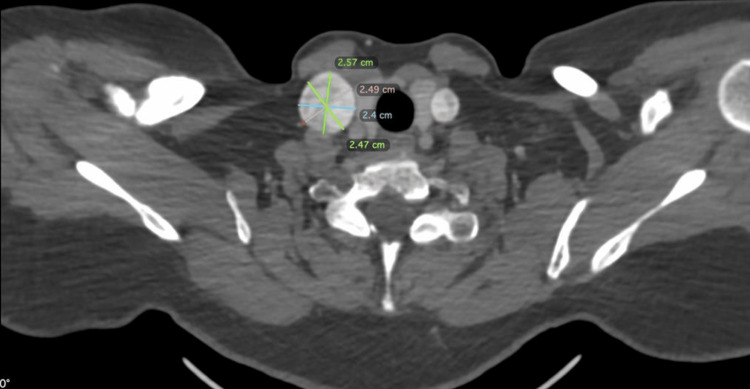
CT angiography confirming right-dominant bilateral jugular phlebectasia Contrast-enhanced CT during the venous phase demonstrated a well-defined fusiform dilatation of the right internal jugular vein, with a maximum diameter of 2.57 cm at the level of the thyroid gland. The vessel shows homogeneous opacification without filling defects or associated vascular anomalies. The left internal jugular vein demonstrates a similar but smaller dilatation.

The combination of clinical and imaging findings established the diagnosis of bilateral jugular phlebectasia, an exceedingly rare condition in adults. The patient remained functionally stable and showed no evidence of complications such as venous thrombosis, rapid enlargement, severe pain, or respiratory compromise. Considering the benign nature of the condition and the potential risks associated with surgical intervention - including vascular injury, residual stenosis, and postoperative thrombosis - a conservative management approach was adopted. This included clinical surveillance, patient education to avoid excessive intrathoracic pressure maneuvers, and intermittent use of nonsteroidal anti-inflammatory drugs for occasional discomfort.

At follow-up, the patient remains clinically stable, with no functional deterioration and no progression in the size of the jugular ectasia.

Written informed consent was obtained from the patient for the publication of this case report and the accompanying clinical images.

## Discussion

Jugular phlebectasia is an uncommon vascular anomaly first described by Harris in 1928 and remains a rare clinical entity [8]. Despite increasing recognition due to advances in imaging techniques, it continues to be underdiagnosed, particularly in adult populations. Bilateral involvement, as observed in the present case, represents an exceptionally rare presentation, accounting for only a small fraction of reported cases [3].

The etiology of jugular phlebectasia remains incompletely understood and is likely multifactorial. Congenital weakness of the venous wall and connective tissue abnormalities have been consistently proposed as primary mechanisms [2]. This is further supported by observations in systemic conditions affecting connective tissue integrity, such as Menkes disease, in which altered lysyl oxidase activity leads to vascular wall fragility and predisposes to venous dilatation [4]. Additional contributing factors include increased intrathoracic pressure and hemodynamic stress, which may exacerbate progressive venous distensibility over time [2]. Acquired mechanisms, such as trauma, mechanical compression, and prior vascular instrumentation, have also been described, although less frequently [7].

Anatomical considerations further explain the predominance of right-sided involvement, as the right internal jugular vein has a more direct anatomical relationship with the superior vena cava and may be subjected to higher hemodynamic pressures [2]. This hemodynamic asymmetry likely contributes to the higher incidence of right-sided and unilateral cases. In contrast, bilateral involvement, as in our patient, suggests a more generalized alteration in venous wall integrity rather than a purely localized phenomenon [7].

Epidemiologically, jugular phlebectasia is predominantly a pediatric diagnosis, with a marked male predominance and right-sided unilateral presentation [4]. Large pediatric series have demonstrated that the condition may remain asymptomatic and underrecognized, with neck masses often noted incidentally or after prolonged evolution [4]. In contrast, adult presentations are significantly less common and may exhibit a different demographic distribution, with a higher prevalence among women [7]. This discrepancy may reflect delayed diagnosis, hormonal influences, or differences in healthcare-seeking behavior. The rarity of a bilateral presentation in adult cases underscores the uniqueness of the present report and expands the clinical spectrum of this condition.

Clinically, jugular phlebectasia typically presents as a soft, compressible, non-pulsatile cervical mass that enlarges during maneuvers that increase intrathoracic pressure, such as the Valsalva maneuver [8]. This dynamic characteristic remains the most important clinical clue and should prompt consideration of vascular etiologies in the differential diagnosis. Other conditions, including laryngocele, pharyngocele, and cystic or mediastinal lesions, may present with similar findings, making accurate clinical assessment essential to avoid misdiagnosis [5].

Imaging plays a central role in diagnosis. Doppler ultrasonography is widely regarded as the first-line modality due to its non-invasive nature and its ability to demonstrate venous dilatation, compressibility, and flow dynamics in real time. It also allows dynamic assessment during Valsalva, which is critical for confirming the diagnosis. CT angiography provides complementary anatomical detail and allows exclusion of associated vascular abnormalities, extrinsic compression, or concomitant anomalies, such as those described in association with other vascular malformations [6]. In the present case, the combined use of Doppler ultrasound and CT angiography enabled accurate diagnosis and exclusion of alternative etiologies [5].

Although jugular phlebectasia is generally considered a benign condition, rare complications have been described, including thrombosis, vocal cord dysfunction, dysphagia, and even neurological manifestations such as Horner’s syndrome due to compression of adjacent structures [7]. However, these complications remain uncommon, and most patients remain asymptomatic or minimally symptomatic throughout their lives.

Management remains a subject of debate due to the rarity of the condition and the lack of standardized guidelines. Nevertheless, current evidence strongly supports a conservative approach in asymptomatic or mildly symptomatic patients, given the benign natural history and low risk of complications [9]. Surgical intervention is generally reserved for patients with significant symptoms, progressive enlargement, cosmetic concerns, or complications such as thrombosis or hemorrhage [7]. Surgical techniques include ligation, excision, or venoplasty; however, these procedures carry inherent risks, including vascular injury, thrombosis, and nerve damage.

In this context, the conservative management adopted in our patient was appropriate, as she remained clinically stable without evidence of complications or functional impairment. This case reinforces the importance of recognizing jugular phlebectasia as a benign and often underdiagnosed condition, particularly in adults, where atypical presentations may lead to unnecessary investigations or interventions.

Furthermore, this report contributes to the limited body of literature on bilateral jugular phlebectasia in adults, highlighting the need for increased clinical awareness and emphasizing the value of dynamic physical examination and targeted imaging in establishing the diagnosis.

## Conclusions

Jugular phlebectasia is a rare and benign vascular condition that should be considered in the differential diagnosis of dynamic cervical masses, particularly those that become evident during the Valsalva maneuver. Although more commonly described in pediatric populations, its occurrence in adults, especially with bilateral involvement, remains uncommon and may lead to diagnostic uncertainty. This case highlights the importance of recognizing its characteristic clinical presentation to prompt appropriate diagnostic evaluation.

Accurate diagnosis relies on the integration of clinical findings with non-invasive imaging modalities, particularly Doppler ultrasonography and CT angiography, which allow precise characterization and exclusion of alternative pathologies. In the absence of significant symptoms or complications, conservative management represents a safe and effective approach. Increased awareness of this condition among clinicians is essential to avoid unnecessary invasive procedures and ensure appropriate patient management.
